# Supplementary material for: A Comparative Study of Experimental Configurations in Synchrotron Pair Distribution Function
Source: Materials (Basel). 2019 Apr 25;12(8):1347. doi: 10.3390/ma12081347 (PMC6515447; doi:10.3390/ma12081347)
Supplement: Supplementary file 1 [file materials-12-01347-s001.pdf]

materials

MDPI

Supplementary Information

A comparative study of experimental configurations in synchrotron pair distribution function

Jesus D. Zea-Garcia, Angeles G. De la Torre, Miguel A. G. Aranda and Ana Cuesta

Table S1. Instrumental parameters for ID22 beamline obtained at Q_{max} 24 Å^{-1} at different data acquisition times.

Beamline ID22	1004 s	400 s	67 s	27 s
Q_{damp} (Å^{-1})	0.0093	0.0094	0.0094	0.0094
Q_{broad} (Å^{-1})	0.0085	0.0085	0.0084	0.0084
R_W (%)	9.3	9.3	9.4	9.5

Table S2. Instrumental parameters for MSPD beamline obtained at Q_{max} 24 Å^{-1} at different data acquisition times.

Beamline MSPD	37 min	37 min × 2 Cycles
Q_{damp} (Å^{-1})	0.0039	0.0039
Q_{broad} (Å^{-1})	0.0085	0.0085
R_W (%)	5.8	5.8

Table S3. Refined unit cell parameters, ADPs, and delta2 values for tricalcium silicate and β-dicalcium silicate samples obtained from the PDF analysis in the 1.4 to 50 Å r-range for ID15A and MSPD data.

Sample (Beamline)		Tricalcium Silicate (ID15A)	Tricalcium Silicate (MSPD)	β-Dicalcium Silicate (ID15A)	β-Dicalcium Silicate (MSPD)
Unit Cell	a (Å)	11.611	11.631	5.499	5.508
	b (Å)	14.182	14.207	6.742	6.755
	c (Å)	13.625	13.653	9.300	9.322
	α (°)	104.8	104.8	-	-
	β (°)	94.5	94.5	94.5	94.5
	γ (°)	90.1	90.2	-	-
ADPs (Å^2)	Ca	0.0054	0.0047	0.0064	0.0061
	Si	0.0050	0.0041	0.0061	0.0059
	O	0.0155	0.0130	0.0172	0.0159
delta2 (Å^2)		1.82	2.60	1.89	2.33

Table S4. Quantitative phase analysis results for the monoclinic tricalcium silicate hydrated paste obtained from the PDF refinements in the r-region from 10 to 25 Å with data collected at ID15A and MSPD. Spdiameter and R_W values are also reported.

Phases	ID15A	MSPD	Expected According to (2) *
Ca_3SiO_5 (wt.%)	25.8	16.0	20#
$Ca(OH)_2$ (wt.%)	22.6	27.1	30.1
$CaCO_3$ (wt.%)	2.5	1.1	-
C–S–H gel (wt.%) **	49.1	55.7	49.9
R_W (%)	31.0	28.5	-

* Theoretical weight percentages of expected hydration products according to reaction (2) showed in the main text. # Assuming a 80% of reaction degree. ** Only includes nanocrystalline defective tobermorite (this number excludes the weight percentage of isolated monolayers of calcium hydroxide and gel pore water).

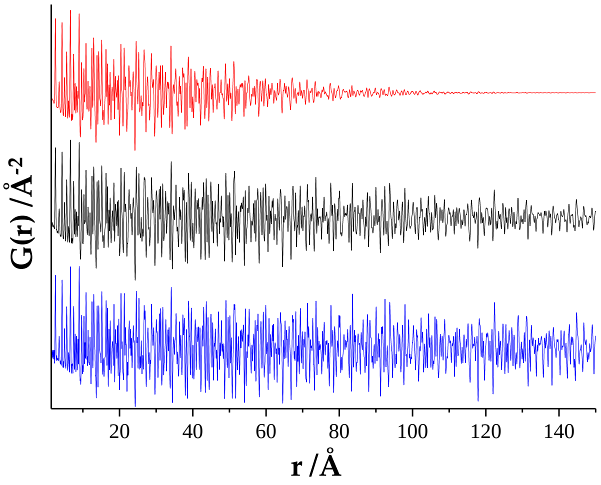

Figure S1. Experimental PDF pattern from 1.35 to 150 Å r-range for the nickel sample with data collected at ID15A (red line), ID22 (black line), MSPD (blue line) beamlines.

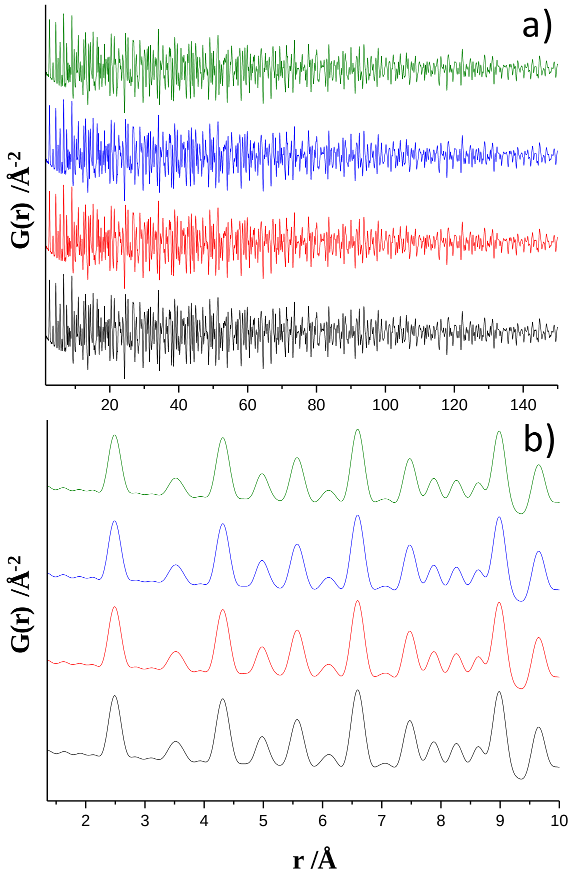

Figure S2. Experimental PDF patterns for the nickel sample with data collected at ID22 at different acquisition times in seconds: 27 (black line), 67 (red line), 400 (blue line), 1004 (green line). (**a**) R-range from 1.35 to 150 Å and (**b**) r-range from 1.35–10 Å.

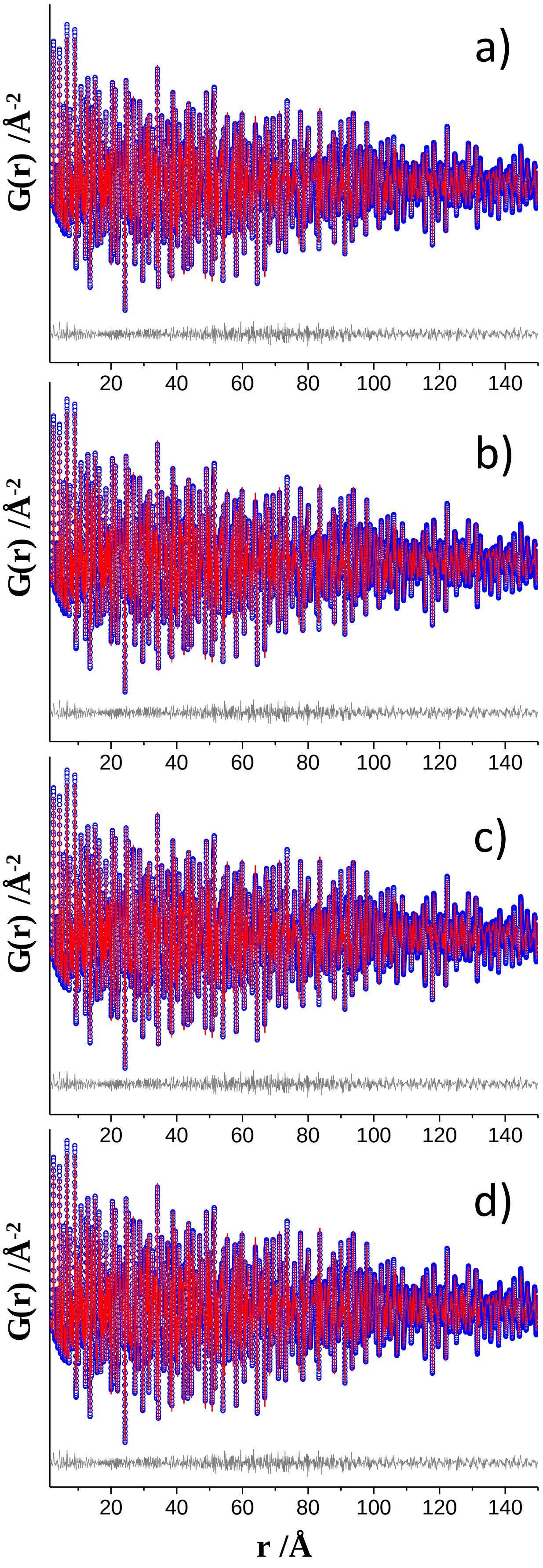

Figure S3. Experimental (blue circles) and fitted (red solid line) PDF patterns for nickel sample with the data collected at ID22 at different acquisition times (**a**) 1004 s, (**b**) 400 s, (**c**) 67 s and (**d**) 27 s in the r-range 1.35 Å to 150 Å. Difference curves are shown as grey lines.

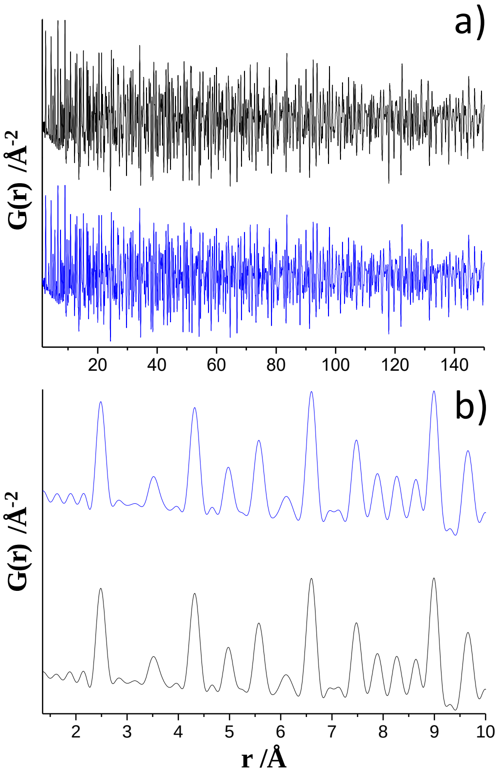

Figure S4. Experimental PDF pattern for the nickel sample with the data collected at MSPD at different acquisition times: 37 min (blue line) and 37 min × 2 cycles (black line). (**a**) R-range from 1.35 to 150 Å and (**b**) r-range from 1.35–10 Å.

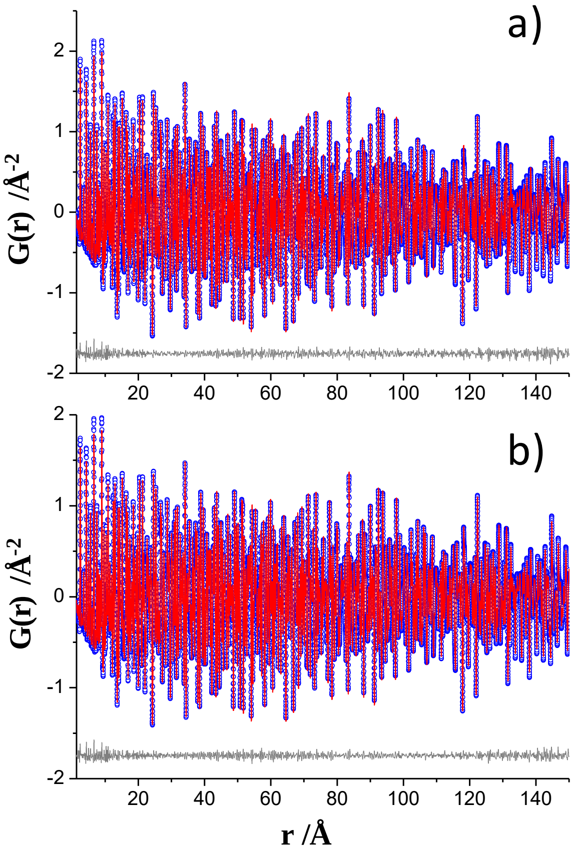

Figure S5. Experimental (blue circles) and fitted (red solid line) PDF patterns for nickel sample with the data collected at MSPD at different acquisition times: (**a**) 37min × 2 cycles and (**b**) 37min in the r-range 1.35 Å to 150 Å. Difference curves are shown as grey lines.

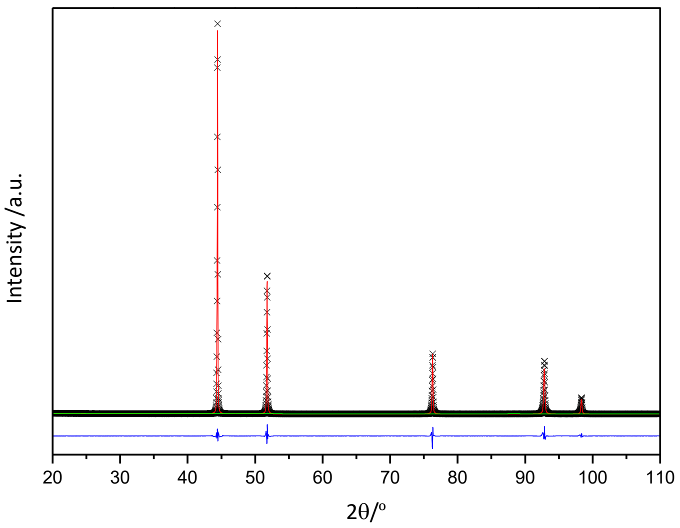

Figure S6. Laboratory X-ray powder diffraction (LXRPD) Rietveld plot (CuKα1 radiation, λ = 1.54059 Å) for nickel sample (ICSD #260169).

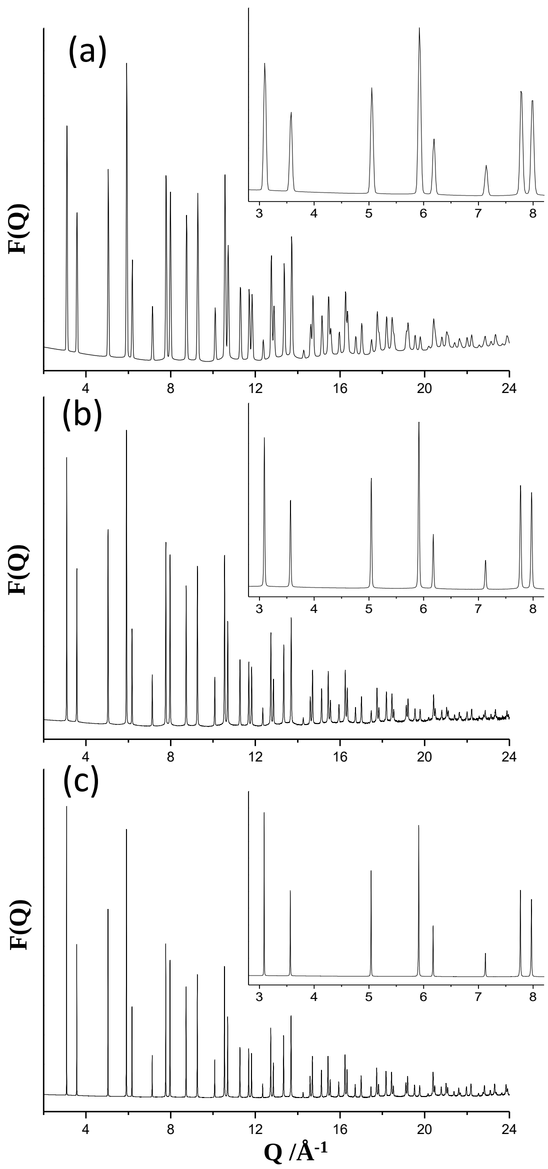

Figure S7. Reduced total scattering structure factor for the data collected at (**a**) ID15A, (**b**) ID22 and (**c**) MSPD. Insets detail the low Q-region for each dataset.

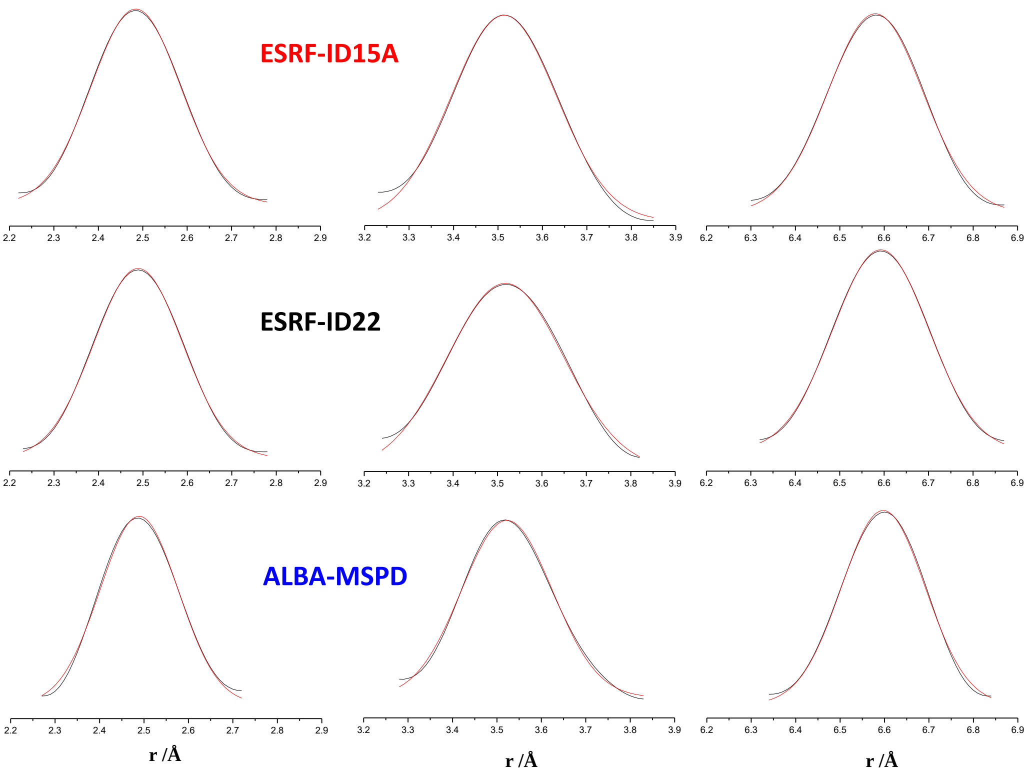

Figure S8. Peak fits for the selected Ni–Ni interatomic distances, at 2.49, 3.63 and 6.58 Å, for the nickel standard in the three experimental configurations.

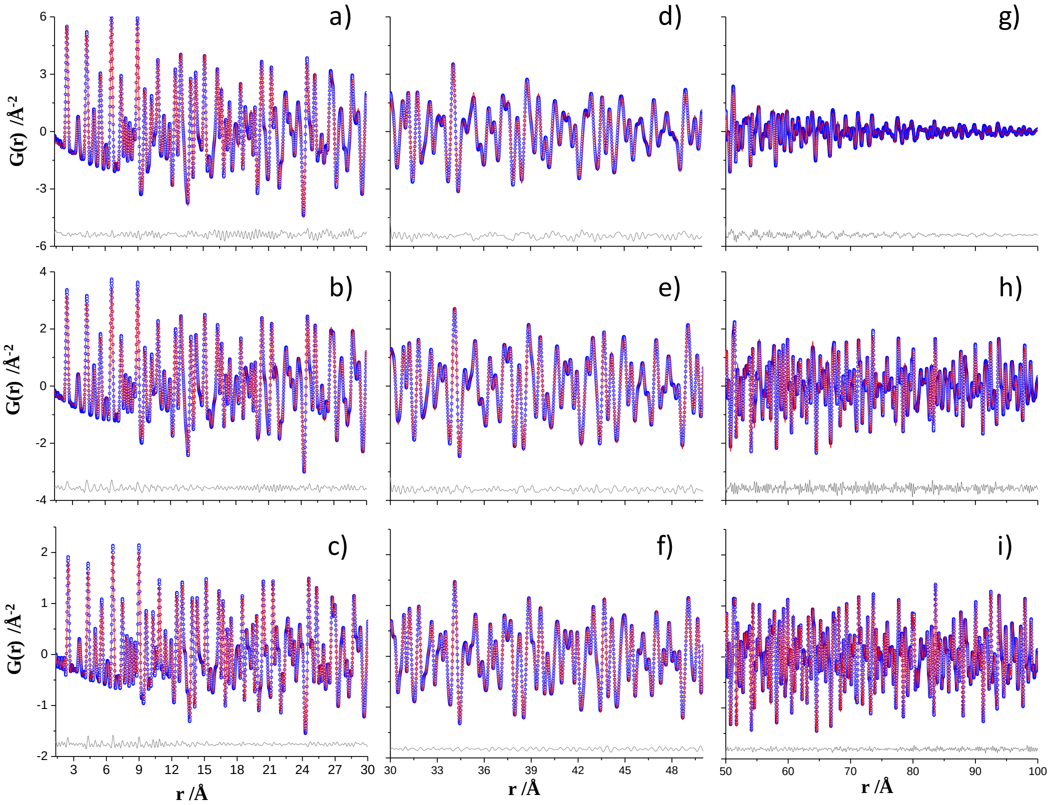

Figure S9. Experimental (blue circles) and fitted (red solid line) PDF patterns for crystalline nickel, Q_{max} = 24 Å^{-1} (**a**) 1.35–30 Å r-range for ID15A data, (**b**) 1.35–30 Å r-range for ID22data, (**c**) 1.35-30 Å r-range for MSPD, (**d**) 30–50 Å r-range for ID15A data, (**e**) 30–50 Å r-range for ID22 data, (**f**) 30–50 Å r-range for MSPD data, (**g**) 50–100 Å r-range for ID15A data, (**h**) 50–100 Å r-range for ID22 data and (**i**) 50–100 Å r-range for MSPD data. Difference curves are shown as grey lines.

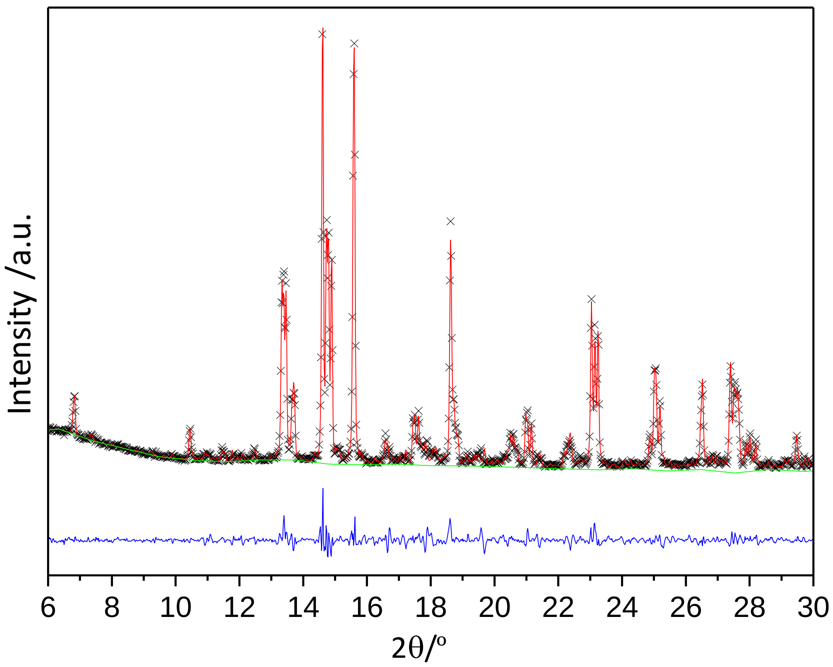

Figure S10. Laboratory X-ray powder diffraction (LXRPD) Rietveld plot (MoKα1 radiation, λ = 0.70932 Å) for triclinic tricalcium silicate (ICSD #162744).

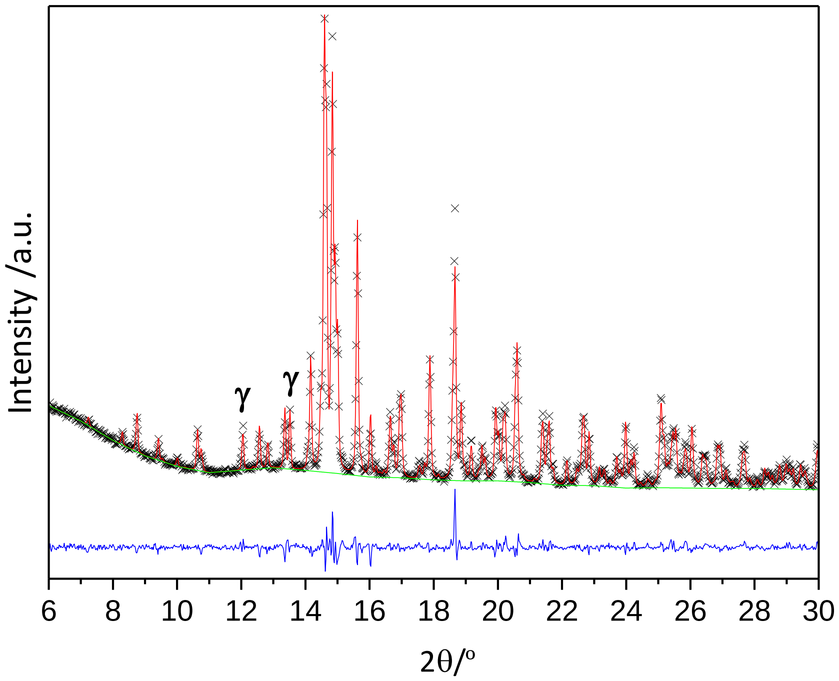

Figure S11. Laboratory X-ray powder diffraction (LXRPD) Rietveld plot (MoKα1 radiation, λ = 0.70932 Å) for the β-dicalcium silicate sample (ICSD #81096) which also contains γ-dicalcium silicate (ICSD #81095). The main diffraction peaks of γ-dicalcium silicate are labeled as "γ".

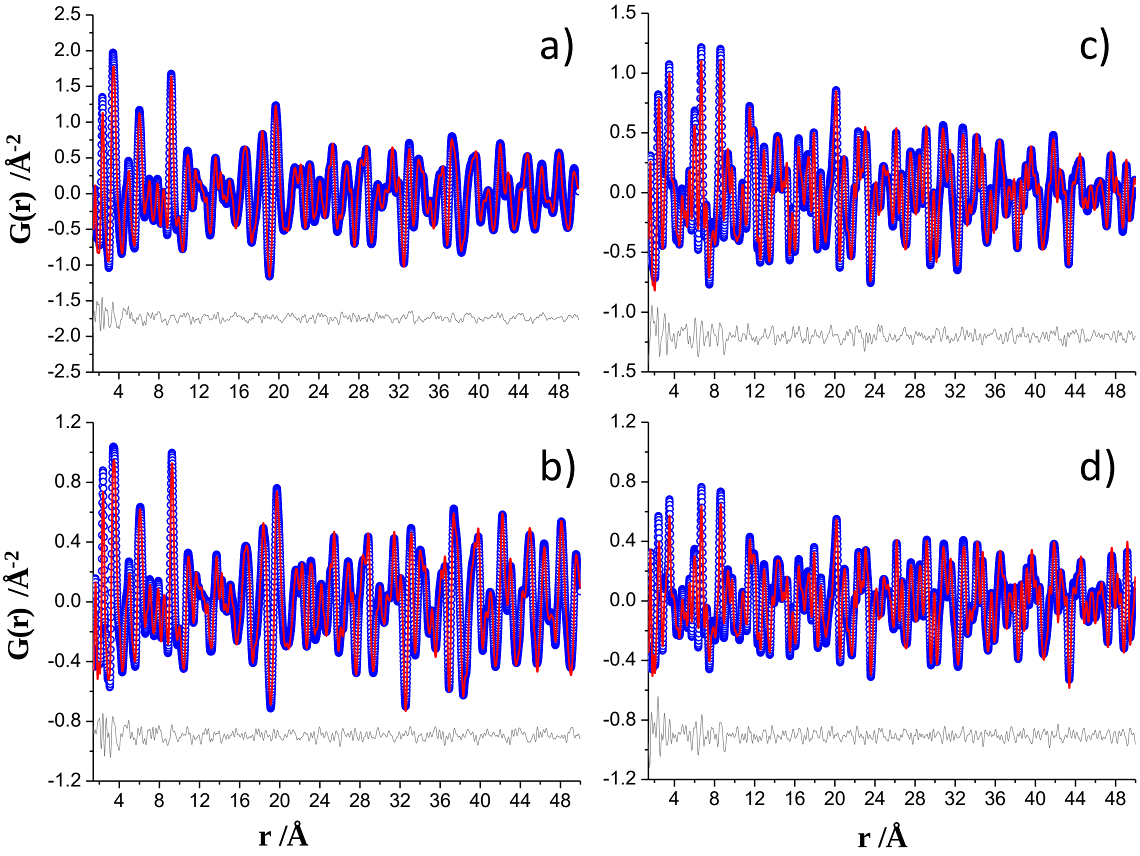

Figure S12. Experimental (blue circles) and fitted (red solid line) PDF in the 1.4–50 Å r-range, Q_{max} = 24 Å^{-1} (**a**) ID15A dataset for triclinic tricalcium silicate, (**b**) MSPD dataset for triclinic tricalcium silicate, (**c**) ID15A dataset for monoclinic dicalcium silicate and (**d**) MSPD dataset for monoclinic dicalcium silicate. Difference curves are shown as grey lines.

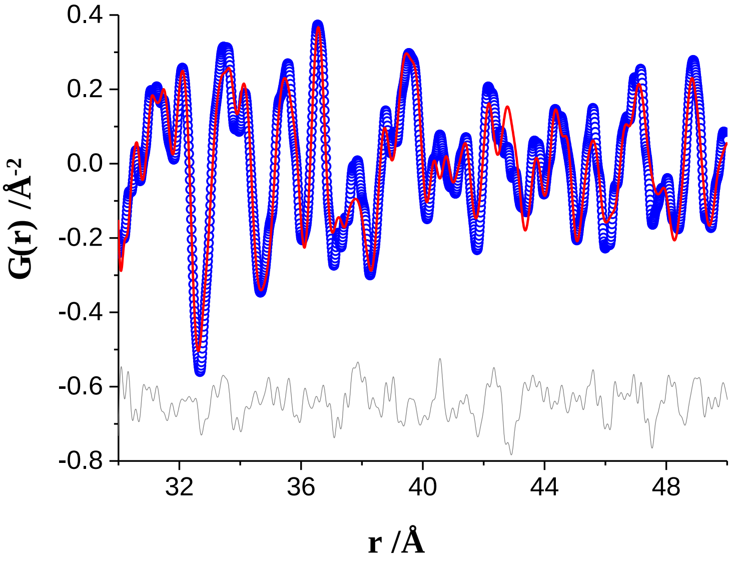

Figure S13. Experimental (blue circles) and fitted (red solid line) PDF for ye'elimite with bassanite hydrated paste, w/s = 1.20 and T = RT for 14 days, in the 30 to 50 Å r-range for ID15A data, Q_{max} = 29 Å^{-1}. Difference curve is shown as grey line.

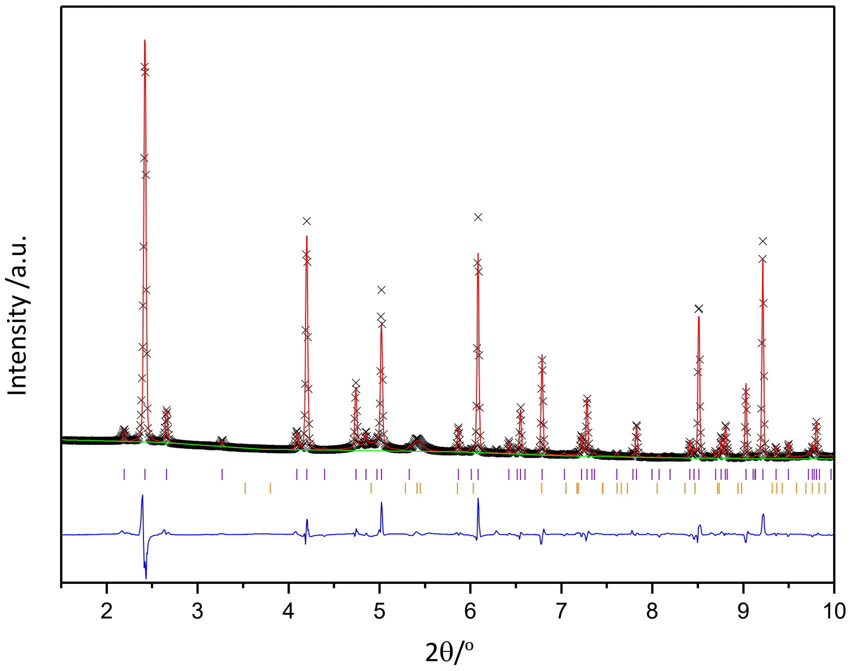

Figure S14. Synchrotron X-ray powder diffraction (SXRPD) Rietveld plot (λ = 0.41236 Å) for ye'elimite with bassanite hydrated paste. Purple tick marks denotes the ettringite reflections and the orange ones denotes gibbsite reflections.
